# Environmental versus extra‐organismal DNA

**DOI:** 10.1111/mec.16144

**Published:** 2021-09-09

**Authors:** Jan Pawlowski, Laure Apothéloz‐Perret‐Gentil, Florian Altermatt

**Affiliations:** ^1^ Department of Genetics and Evolution University of Geneva Geneva Switzerland; ^2^ ID‐Gene ecodiagnostics Plan‐les‐Ouates Switzerland; ^3^ Institute of Oceanology Polish Academy of Sciences Sopot Poland; ^4^ Department of Aquatic Ecology Eawag, Swiss Federal Institute of Aquatic Science and Technology Zurich Switzerland; ^5^ Department of Evolutionary Biology and Environmental Studies Faculty of Science University of Zurich Zurich Switzerland; ^6^ Research Priority Programme Global Change and Biodiversity (URPP GCB) University of Zurich Zurich Switzerland

We are very pleased that our opinion paper “Environmental DNA: What's behind the term?” (Pawlowski et al., 2020) stimulated a lively discussion and we are grateful for the comments on proposed terminology (Rodriguez‐Ezpeleta et al., 2021). The clarity of scientific terms is essential for both fundamental and applied research and any debate on this issue is very important, especially in the early days of a new field. A major requirement of clarity is that terminology refers to measurable and implementable classifications. To recall the context of this debate, the aim of our paper was to restore a broad definition of environmental DNA (eDNA) as referring to all organisms present in environmental samples, including both macrobial and microbial organisms (Taberlet et al., 2012).

We are very glad that our proposition to adopt a broad definition of eDNA was accepted by Rodriguez‐Ezpeleta et al. (2021). However, we cannot agree with their opinion that our two‐level terminology is oversimplistic. Our terminology refers to the two basic steps of any eDNA metabarcoding study, defining first the material (i.e., environmental sample) taken for DNA extraction and second the taxonomic group targeted by PCR (polymerase chain reaction). This may be simple but is also directly and clearly applicable, thereby clarifying the aims and targets of eDNA studies in general. We privilege the choice of material and method over the potential outputs of a study. According to our view, even if sediment samples are used as a source of information about present or past surface plankton, these are still sediment eDNA studies (e.g., Monchamp et al., 2018; Morard et al., 2017). Similarly, if taxon‐specific PCR primers also amplify other taxa, the target taxon should be mentioned, rather than PCR byproducts (e.g., Mächler et al., 2019).

It is important to highlight that our proposed classification remains open to a more detailed specification of the study of eDNA. We think that targeting extra‐organismal DNA corresponds to such complementary information and this information can be included in the description of the study. As correctly emphasized by Rodriguez‐Ezpeleta et al. (2021), the ecological interpretation of extra‐organismal DNA data must consider many factors specific to this type of DNA. Nevertheless, in practice such a distinction at best concerns only those organisms over a certain size. As shown in figure 1 of Rodriguez‐Ezpeleta et al. (2021), there is overlap over at least six orders of magnitude in size between intra‐ and extra‐organismal DNA, and even they conclude that “it is currently impractical to separate and independently analyse organismal and extra‐organismal DNA.” Given the continuous occurrence and transition of DNA from living organisms, to within tissues or cells (living or dead), to organelles and truly free DNA, we also think such separation is methodologically challenging if not impossible, and thus not directly applicable. While smaller‐sized organisms (microbes or small animals such as rotifers) may be indeed often sampled in their living state, they can still also be recorded through DNA from degraded cells or organisms. By analogy, large organisms, such as mussels, may be largely recorded by extra‐organismal DNA, but the occurrence of veliger larvae in water eDNA samples may go unnoticed and not be separable. Indeed, the complex mixture of different‐origin (or “types” of) DNA may be difficult to resolve, and we recommend sticking to the directly applicable, technical terminology proposed by us. We fully understand the importance of eDNA for the detection and monitoring of aquatic vertebrates, especially fish and amphibians, and we recognize the need to assess the specific biases and types of noise related to its primarily extra‐organismal character. In this particular case, the origin of eDNA is *selbstverständlich*, so it is unlikely that the meaning of the term might lead to confusion.
